# MicroRNAs (miRNAs): Novel potential therapeutic targets in colorectal cancer

**DOI:** 10.3389/fonc.2022.1054846

**Published:** 2022-12-14

**Authors:** Ying Yang, Wen-Jian Meng, Zi-Qiang Wang

**Affiliations:** Colorectal Cancer Center, Department of General Surgery, West China Hospital, Sichuan University, Chengdu, China

**Keywords:** colorectal cancer, MicroRNAs, drug resistance, metastasis, signaling pathways, therapeutic targets

## Abstract

Colorectal cancer (CRC) is the most common malignant tumor and one of the most lethal malignant tumors in the world. Despite treatment with a combination of surgery, radiotherapy, and/or systemic treatment, including chemotherapy and targeted therapy, the prognosis of patients with advanced CRC remains poor. Therefore, there is an urgent need to explore novel therapeutic strategies and targets for the treatment of CRC. MicroRNAs (miRNAs/miRs) are a class of short noncoding RNAs (approximately 22 nucleotides) involved in posttranscriptional gene expression regulation. The dysregulation of its expression is recognized as a key regulator related to the development, progression and metastasis of CRC. In recent years, a number of miRNAs have been identified as regulators of drug resistance in CRC, and some have gained attention as potential targets to overcome the drug resistance of CRC. In this review, we introduce the miRNAs and the diverse mechanisms of miRNAs in CRC and summarize the potential targeted therapies of CRC based on the miRNAs.

## Introduction

Colorectal cancer (CRC) is the third most frequent cancer and the second leading cause of cancer death, with an estimated more than 1.9 million new cases and 935,000 deaths worldwide in 2020 (1). The incidence of CRC is steadily increasing year by year in many countries, with a trend of younger age onset (1, 2). At present, the treatment of CRC mainly includes surgery, radiotherapy, chemotherapy, immunotherapy and targeted therapy. However, drug resistance and recurrence after therapy are still the major obstacles to effective anticancer therapy for CRC (3–5), and it is reported that approximately 50% of CRCs are resistant to 5-Fu-based chemotherapy regimens (6). In addition, immunotherapy (checkpoint blockade therapies) is currently considered to be only effective for a proportion (approximately 10-15%) of mismatch-repair-deficient (dMMR) CRC (7–9). The prognosis of CRC was negatively correlated with the progression of tumor stage. Although these treatments can improve the survival rate of CRC patients, the 5-year survival rate for those diagnosed with distant-stage disease remains poor (only approximately 14%) (2). Therefore, it is necessary to find new therapeutic strategies that can effectively treat CRC or improve the drug resistance of CRC to prevent CRC relapse and improve the prognosis of CRC patients.

MicroRNAs (miRNAs/miRs) are a category of short, noncoding, highly conserved and single-stranded RNAs that regulate gene expression at the posttranscriptional level by mRNA degradation or silencing (10–12). MiRNAs are not only critical for regulating normal physiological activities in various biological processes, such as cell development, metabolism, proliferation and apoptosis, but also play an important role in the progression of cancer (13, 14). In 2003, the reduction in miRNAs was reported to be closely related to CRC (15). Since then, research focusing on the effects of miRNAs on CRC has gradually shown that aberrant expression of miRNAs is associated with CRC progression, including tumor formation, metastasis, and drug resistance (16–19). For instance, Li et al. (20)reported that the expression of miR-186-5p in CRC cell lines (HT116, H29, SW620 and LoVo) was lower than that in the normal colonic epithelial cell line NCM460. Moreover, the high expression of miR-186-5p can inhibit the proliferation, epithelial-to-mesenchymal transition (EMT), and metastasis of the CRC cell line LoVo by targeting inhibition of ZEB1. Jin et al. (21) found that the expression of miRNA-30a was significantly increased in CRC tissues compared with normal colorectal tissues, and the expression level of miRNA-30a was inversely correlated with the invasiveness of CRC cell lines. Therefore, miRNAs are expected to be potential novel therapeutic targets for CRC.

Therefore, in this article, we will review the latest research progress on the involvement of miRNAs in the occurrence and development of CRC. This review also discusses the current understanding of CRC drug resistance-related miRNAs and their underlying molecular mechanisms. Furthermore, we highlight the potential of miRNAs as CRC therapeutic targets.

### Biosynthesis and mode of action of miRNA

**Figure 1:**
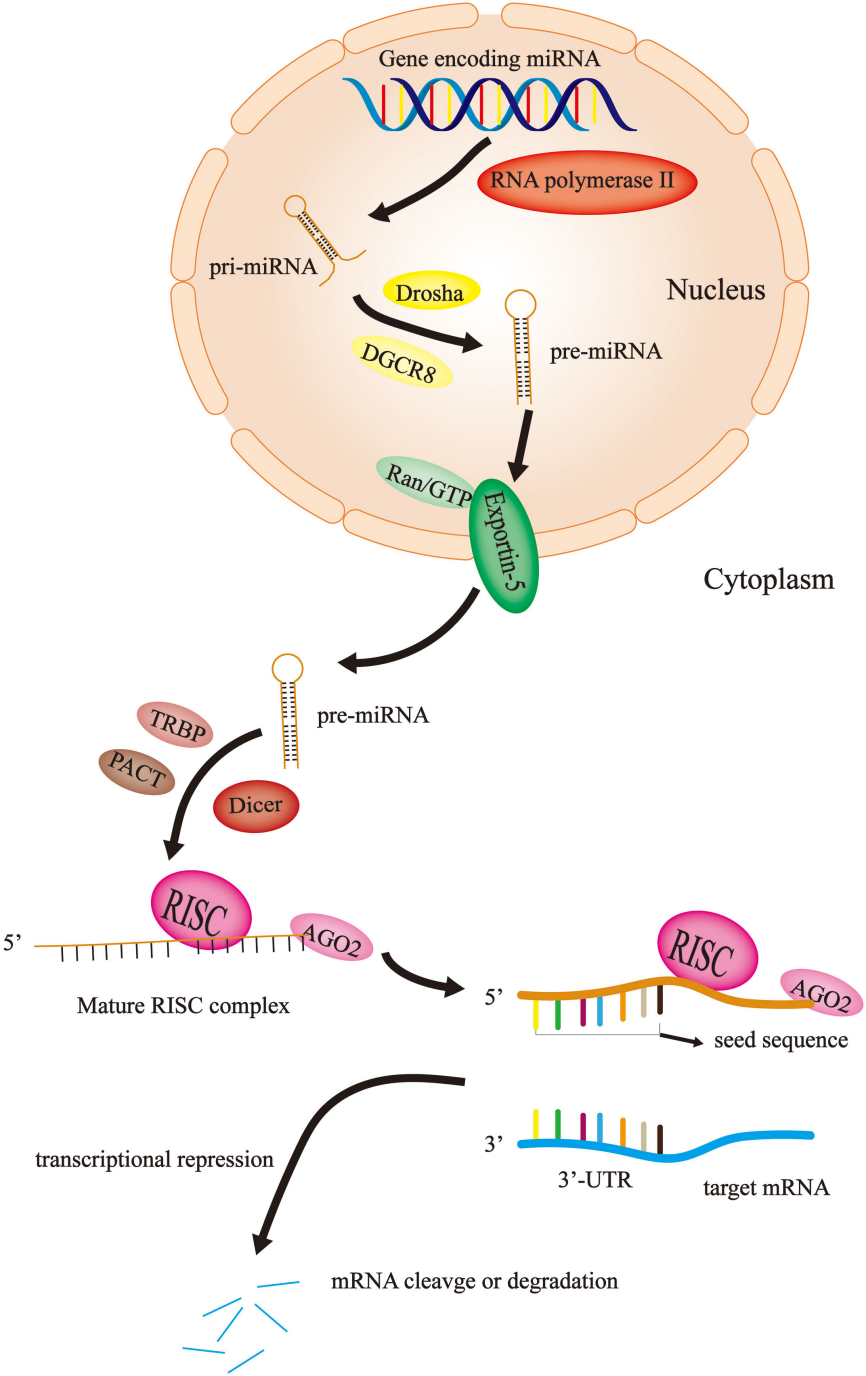
miRNA biosynthesis and mechanism of action. In the nucleus, the miRNA gene is transcribed by RNA polymerase II into pri-miRNA, which become pre-miRNA with the help of Drosha and DGCR8. Then, the Ran/GTP/Exportin-5 complex delivers the pre-miRNA to the cytoplasm. Here, with the assistance of Dicer, TRBP and PACT, the pre-miRNA is disassembled into single strands and combined with AGO2 and RISC to become a mature RISC complex. Finally, the RISC complex binds to the target mRNA to cause mRNA degradation or translational repression.

miRNAs are short, noncoding RNAs that are not translated into proteins, with a length of approximately 22 nucleotides (22). The first animal microRNA was lin4, called “small molecule sequential RNA (stRNA)”, which was found in Caenorhabditis elegans in 1993 (23). It was not until 2001 that a large number of functional microRNAs with common characteristics were found in many species, which were named miRNAs (24–26). Until recently, the exploration of the roles and mechanisms of this noncoding RNA in biological activities was in progress. Currently, the majority of studies agree that miRNA is derived from a primary miRNA transcript (pri-miRNA). Firstly, the miRNA gene is transcribed into a large pri-miRNA containing multiple hairpin-loop structures by RNA polymerase II in the nucleus (27). The pri-miRNA typically contains thousands of nucleotides; subsequently, the pri-miRNA is converted into a shorter hairpin-shaped precursor miRNA (pre-miRNA) containing approximately 70 nucleotides by the Drosha enzyme (one type of RNAseIII) and DGCR8 in the nucleus (28–30). Pre-miRNA is transported from the nucleus to the cytoplasm by the Ran/GTP/Exportin-5 complex (31, 32); the pre-miRNA is cleaved and processed to form a double-stranded miRNA of approximately 22 nucleotides in length in the cytoplasm with the help of the Dicer enzyme (another type of RNAseIII), TRBP and PACT (33–35). Finally, one strand of the double-stranded miRNA is bound to AGO2 and loaded onto the RNA-induced silencing complex (RISC) to become the RISC complex, while the other strand is degraded (36). The mature RISC complex binds to target mRNAs with complementary sites, resulting in translational inhibition or degradation of target mRNAs (37) (**Figure 1**).

Initially, it was not thought that these noncoding miRNAs could have a severe impact on human health and even lead to the occurrence and progression of cancer. With the continuous exploration of miRNAs, humans have gradually changed this view. Currently, it is widely assumed that miRNAs carrying RISC recognize the binding site on the 3’-UTR of the target gene mRNA through its seed sequence (nucleotides 2-8 at the 5-terminal). This process produces two effects: transcriptional repression and mRNA cleavage or degradation (38) (**Figure 1**). In 2002, Calin et al. (39)showed that miRNA genes (miR-15 and miR-16) at 13q14 in chronic lymphocytic leukemia are usually deleted or downregulated by detailed deletion and expression analysis. This study suggests that miRNAs may play an important role in tumorigenesis and tumor progression. Thus, since then, an increasing number of studies have demonstrated that the aberrant expression of miRNAs is associated with cancer progression, including CRC (40–42).

Intestinal microbiome is considered as a key participant in CRC immune regulation and tumor promoting microenvironment, because bacteria from different intestinal sources can induce tumor growth. In recent years, studies have found that the cross-talk between gut microbes and the host in tumor cell metabolism is largely achieved by regulating the level of miRNAs (43, 44). Intestinal microbiota can affect the expression of miRNA, and the abnormal expression of miRNA subsequently activates signal pathways and regulates various aspects of tumor pathobiology in CRC (45). The miRNA in the tumor microenvironment alters the composition of the intestinal microbiota by influencing the gene expression of the microbiota and transferring the metabolites secreted by cancer (46). In conclusion, this interaction will eventually create a favorable microenvironment for tumor cells, including angiogenesis, immune escape and microbiota composition. Meanwhile, the two-way interaction between host and gut microbiome mediated by miRNAs brings new complexity to miRNAs research.

### The role of miRNAs in CRC

There is abundant evidence that miRNAs and their biogenesis mechanisms are related to the occurrence and development of CRC. Compared to normal tissues, miRNAs are frequently dysregulated in tumors. It suggests that the aberrant expression of miRNAs is closely related to the progression, metastasis and drug resistance of CRC.

## miRNAs involved in CRC progression and metastasis

The progression and metastasis of CRC are the result of a multistep process involving multiple genetic and epigenetic alterations of oncogenes and tumor suppressor genes over time. In addition, metastasis is also a major cause of poor prognosis in CRC patients. In recent years, substantial data have identified important roles for miRNAs in numerous regulators involved in CRC pathogenesis and metastasis (47, 48). It is being increasingly regarded that miRNA regulation of CRC progression and metastasis occurs by various mechanisms, such as influencing signaling pathways, EMT, and angiogenesis.

There are many signaling pathways mediated by miRNAs in CRC cells that play an important role in the progression and metastasis of CRC (**Table 1**). As a regulatory pathway regulating colorectal development, hyperactivation of the Wnt/β-catenin pathway is found in more than 90% of CRC cells. The loss or inactivation of adenomatous polyposis coli (APC, a key negative regulator of the canonical Wnt signaling pathway) and the overactivation of the Wnt/β-catenin signaling pathway are considered to be the key processes in the initiation of CRC (83, 84). Zheng et al. (49)aimed to investigate the tumorigenic role of miR-490-3p in CRC. They reported that downregulation of miR-490-3p promoted CRC progression by activating the classic Wnt/β-catenin signaling pathway. MiR-224 has been found to directly target the GSK3β and SFRP2 genes to activate Wnt/β-catenin signaling and direct the nuclear translocation of β-catenin in CRC. Furthermore, miR-224 upregulation promoted CRC cell proliferation. Knockdown of miR-224 attenuated the effects of Wnt/β-catenin on the metastasis and proliferation of CRC cells (50). Similarly, miR-452 enhanced the proliferation and metastasis of CRC cells by activating the Wnt/β-catenin signaling pathway *via* directly targeting GSK3β. While knockdown of miR-452 was found to restore the expression of GSK3β and inhibit Wnt/β-catenin-mediated cell metastasis and cell proliferation (51). The expression of miR-494 was significantly increased in CRC, with a negative correlation to APC expression in CRC tissues. And up-regulation of this miR-494 has also been found to enhance cell proliferation and tumorigenesis of CRC by suppressing the expression of APC (52). As a member of the BTB/POZ/zinc finger (ZF) family of transcription factors, BCL6 has been shown to target multiple functional signaling pathways, including the Wnt/β-catenin signaling pathway. In addition, Sun et al. (53) suggested that BCL6 was a mediator of miR-144-3p in regulating CRC cell proliferation and cell cycle arrest. They reported that miR-144-3p inhibited cell proliferation and the G1/S phase transition of CRC cells by targeting BCL6 *via* inhibition of Wnt/β-catenin signaling. As a negative regulator of Wnt/β-catenin signaling, miR-377-3p was shown to inhibit CRC cell growth *in vitro* and *in vivo* by targeting XIAP and ZEB2 (54). The PI3K/AKT signaling pathway is an important signaling pathway that controls the growth and metastasis of CRC (85). Jia et al. reported that miR182/-135b promoted CRC progression by targeting ST6GALNAC2 *via* regulation of the PI3K/AKT pathway (55). miR-7 conferred its tumor-suppressing function in CRC by inhibiting the activation of the PI3K/AKT pathway by downregulating the miR-7 potential target TYRO3 (56). Therefore, targeting these signaling pathways mediated by miRNAs that regulate the progression and metastasis of CRC may become a promising therapeutic strategy for targeting CRC.

**Table 1:** A list of representative miRNAs identified in CRC that are associated with the progression/metastasis of CRC.

miRNA	mechanism	Targets/Regulators	Function	Reference
miR-490-3p	Wnt/β-catenin signaling pathway	FRAT1	Inhibition of CRC progression	(49)
miR-224	Wnt/β-catenin signaling pathway	GSK3β and SFRP2	Promotion of aggressive phenotype of CRC	(50)
miR-452	Wnt/β-catenin signaling pathway	GSK3β	Promotion of aggressive phenotype of CRC	(51)
miR-494	Wnt/β-catenin signaling pathway	APC	Promotion of CRC progression	(52)
miR-144-3p	Wnt/β-catenin signaling pathway	BCL6	Inhibition of CRC cell proliferation and G1/S phase transition	(53)
miR-377-3p	Wnt/β-catenin signaling pathway	XIAP and ZEB2	Inhibition of CRC cell growth	(54)
miR182/-135b	PI3K/AKT pathway	ST6GALNAC2	Promotion of CRC progression	(55)
miR-7	PI3K/AKT pathway	TYRO3	Inhibition of the proliferation, migration and invasion of CRC	(56)
miRNA-34a	STAT3	PPP1R11	Inhibition of tumor metastasis and EMT	(56)
miR-302c	EMT	transcription factor AP-4	Inhibition of metastasis of CRC	(57)
miR-30a	EMT	TM4SF1	Inhibition of migration and invasion of CRC	(58)
miRNA-9	EMT	TM4SF1	Inhibition of migration and invasion of CRC	(59)
miRNA-206	EMT	TM4SF1	Inhibition of migration and invasion of CRC	(60)
miR-590-5p, miR-1249, miR-622	angiogenesis	VEGF-A	Inhibition of angiogenesis and metastasis of CRC	(61–66)
miR-150-5p	angiogenesis	VEGF-A	Inhibition of CRC cell proliferation, migration, invasion and angiogenesis	(67)
miR-125	–	VEGF	Inhibition of CRC cell growth	(68)
miR-19a-3p	WNT/β-catenin signaling pathway	FOXF2	Inhibition of CRC cell proliferation	(69)
miR-501-3p	WNT/β-catenin signaling pathway	APC	Promotion of CRC cell proliferation and stemness	(70)
miR-346-5p	WNT/β-catenin signaling pathway	FBXL2	Inhibition of CRC cell proliferation	(71)
miR-552	WNT signaling pathway	p53 tumor suppressor	Promotion of CRC cell proliferation	(72)
miR-137-3p	EMT	–	Inhibition of CRC cell migration	(73)
miR-10a	EMT	–	Inhibition of CRC metastasis	(74)
miR-363-3p	EMT	Sox4	Inhibition of CRC metastasis	(75)
miR-128	Akt-p53-cyclin pathway	RPN2	Inhibition of cell proliferation and migration	(76)
miR-1236-3p	EMT	DCLK3	Inhibition of the proliferation, invasion, and migration of colon cancer cells	(77)
miR-212	AKT/mTOR signaling pathway	PIK3R3	Inhibition of CRC cell viability and invasion	(78)
miR-146b-5p	–	TRAF6	Promotion of CRC initiation and tumorigenesis	(79)
miR-410-3p	NF-κB pathway	ZCCHC10	Promotion of migration, invasion and EMT of CRC	(80)
miR-206	c-Met/AKT/GSK-3β pathway	–	Inhibition of the proliferation, migration and invasion of CRC	(81)
miR-195-5p	–	NOTCH2	Inhibition of CRC cell proliferation, clone formation, migration, and invasion	(82)
miR-224	–	SMAD4	Promotion of CRC metastasis	(19)

It is widely accepted that epithelial–mesenchymal transition (EMT) is the transition from epithelial cells to mesenchymal cells during the progression and metastasis of malignancy. And EMT is known to be associated with a wide array of malignant behaviors of CRC, including tumorigenicity and metastasis (82). There is growing evidence that miRNAs play crucial roles in regulating the phenotype of EMT (86). Ding et al. (73) illustrated that miR-137-3p could attenuate CRC cell migration by regulating a KDM1A-dependent EMT process. Li et al. (87) reported that miRNA-34a inhibits tumor metastasis and EMT by reducing the expression of PPP1R11 to prevent the activation of STAT3. Low expression levels of miR-302c was found to significantly correlate with advanced tumor stage, lymph node metastasis and deeper tumor invasion. And miR-302c inhibit EMT and metastasis of CRC by targeting the transcription factor AP-4 (57). Transmembrane 4 L six family member 1 (TM4SF1) is a direct target gene of miRNAs in CRC cells and is involved in the regulation of EMT progression in CRC. MiR−30a, which is involved in the EMT of CRC, was shown to suppress malignant behaviors of CRC, including migration and invasion, by directly targeting oncogenic TM4SF1 (58). Similarly, miRNA-206 and miRNA-9 could directly target TM4SF1, thus suppressing EMT of CRC cells and leading to the suppression of cell proliferation, migration, and invasion in CRC cells (59, 60).

One of the characteristics of tumor growth is angiogenesis, which fosters tumorigenesis and metastasis by supplying oxygen and diffusible nutrients as well as releasing proangiogenic chemicals. Angiogenesis is integral to the development and progression of CRC, and it is crucial to the growth and metastasis of CRC (61). There are many angiogenic factors that control angiogenesis, including vascular endothelial growth factor (VEGF) and hypoxia-inducible factor 1 (HIF-1). The overexpression of miR-145-5p suppressed RHBDD1 through suppression of the EGFR-associated signaling pathway (EGFR/Raf/MEK/ERK cascades), which in turn inhibited the growth, invasion and migration of CRC cells (62). As a key VEGF receptor, VEGF-A is involved in angiogenesis and stimulates the germination of prevascular endothelial cells, which results in the development of new vasculature. MiR-590-5p was found to suppress the angiogenesis and metastasis of CRC by modulating VEGF-A (63). Similarly, miR-1249 and miR-622 can also inhibit CRC angiogenesis by regulating the level of VEGF-A, thereby inhibiting CRC growth and metastasis (64, 65). Additionally, miR-520a serves as a direct target of VEGF-A, and ATAD2 can suppress VEGF-A production by elevating the expression of miR-520a, hence inhibiting angiogenesis in CRC (66). *In vitro* and *in vivo*, miR-150-5p inhibited the ability of CRC cells to proliferate, migrate, invade, and undergo angiogenesis. This inhibitory impact could be reversed by transfecting a plasmid encoding the VEGF-A (67). HIF-1 is a critical regulator of VEGF and one of the key molecules that mediates the growth of CRC (88, 89). Additionally, numerous miRNAs have been identified as having a role in the regulation of HIF-1 on the angiogenesis and development of CRC (90, 91). MiR-148a inhibited CRC angiogenesis and reduced the risk of early recurrence of CRC by regulating the level of pERK/HIF-1α (92).

In conclusion, miRNAs play an important regulatory role in the biological behavior of CRC. MiRNAs affect the self-renewal, proliferation, differentiation, metastasis and other biological behaviors of CRC by regulating related molecules of various signaling pathways, EMT or angiogenesis. And miRNAs may have broad prospects in clinical application. Further exploration of key miRNAs and their regulatory mechanisms related to the characteristics of CRC will provide more references for future CRC targeted therapy.

## MiRNAs and drug resistance in CRC

Resistance to anticancer therapy is one of the major barriers to the successful treatment of CRC. It was reported that there were approximately 80% of responders may develop drug resistance (93). The mechanism of drug resistance acquired by CRC has been continuously explored, and it is believed that the drug resistance of CRC is associated with multiple factors, such as the cancer stem cells and tumor microenvironment of CRC (94, 95). However, no single viewpoint can explain the drug resistance of all CRC patients. In recent years, an increasing number of studies have shown that miRNAs are involved in the drug resistance of CRC by regulating autophagy, the cell cycle, important signaling pathways and efflux pumps (**Table 2**).

**Table 2:** Effects of miRNAs on anticancer regimens in CRC *in vitro* and *in vivo* studies.

MiRNAs	Effect of miRNA on resistance	Anticancer regimens	Reference
miR-145	up	5-FU	(96)
miR-195-5p	up	cisplatin	(97)
miR-92b-3p	up	MDR	(98)
miR-27b-3p	down	OXA	(99)
miR-577	up	5-FU	(100)
miR-506	down	OXA	(101)
miR-199b-3p	up	5-FU	(102)
miR-200b-3p	down	OXA	(103)
miR-454-3p	up	OXA	(104)
miR-135b,miR-182	up	5-FU	(105)
miR-543	up	5-FU	(106)
miR-455-5p	down	5-FU	(107)
miR-148a	down	cisplatin	(108)
miR-139-5p	down	5-FU	(109)
miR-128-3p	down	OXA	(110)

Autophagy is a type of programmed cell death different from apoptosis. Autophagy is the process of transporting damaged, denatured or aged proteins and organelles in cells to lysosomes for digestion and degradation. On the one hand, autophagy is a physiological process and a defense mechanism of cells in adverse environments. On the other hand, the occurrence and development of autophagy is also closely related to the drug resistance of tumors (111, 112). In recent years, it has been reported that miRNAs regulate the drug resistance by modulating autophagy (113). Despite tremendous progress in anticancer therapy, 5-FU-containing regimens remain one of the most commonly used and effective treatment regimens for CRC. Furthermore, evidence suggests that miRNAs may be involved in the chemoresistance of CRC cells to 5-FU. Zhao et al. (96) found significantly low expression of miR-145 and p53, whereas the expression of activating transcription factor 4 (ATF4) histone deacetylase 4 (HDAC4) increased considerably by RT-qPCR and Western blot analysis. They further found that ATF4-regulated miR-145 enhanced CRC tumorigenesis and the resistance to 5-FU *via* regulating the HDAC4/p53 axis. Oxaliplatin (OXA) is another crucial component of the combinatorial chemotherapeutic standard of CRC. And OXA resistance is also another major obstacle to effective chemotherapy in CRC patients. In OXA-resistant cell lines (SW480-OxR and HCT116-OxR), the expression of miR-27b-3p was significantly decreased compared to that in the corresponding parental cells. miR-27b-3p was able to inhibit autophagy in CRC cells by suppressing the expression of ATG10 at the posttranscriptional level. Meanwhile, miR-27b-3p could increase the sensitivity of CRC cells to OXA *in vivo* and *in vitro* by regulating the level of autophagy (99). However, there are still few studies on miRNAs affecting CRC drug resistance by regulating autophagy levels, and more high-quality studies are needed in the future to confirm this view.

The cell cycle is the process of DNA replication and cell division and includes four stages: G1, S, G2 and M (114). Studies have shown that impaired cell cycle regulation is the key mechanism that promotes drug resistance in cancer. However, miRNAs may facilitate the resistance of cancer cells to chemotherapy by affecting the cell cycle (115). Zhao et al. (98)found that miR-92b-3p was highly expressed by CRC HCT8/T cells and that knockdown of miR-92b-3p may attenuate the resistance of MDR HCT8/T cells to chemotherapy *in vitro* and *in vivo*. They further demonstrated that miR-92b-3p regulated the sensitivity of CRC cells to chemotherapeutic drugs by regulating the cell cycle and apoptosis (via directly targeting cyclin-dependent kinase inhibitor 1C and suppressing its expression). The expression of miRNA-375-3p was significantly decreased in CRC cell lines, and the expression level of miRNA-375-3p was proportional to the sensitivity of CRC cells to 5-FU. It was confirmed to enhance the sensitivity of CRC cells to 5-FU by inducing apoptosis and cycle arrest of CRC cells (116). The expression of miR-577 was found to be significantly increased in 5-fluorouracil (5-FU)-resistant SW480 cells (SW480/5-FU). And Jiang et al. revealed that miR-577 inhibited tumor growth and enhanced 5-FU sensitivity in SW480/5-FU cells by inducing G0/G1 cell cycle arrest in CRC cells (100).

Many signaling pathways have been shown to be involved in tumor chemoresistance. In recent years, there has also been growing evidence that miRNAs regulate the resistance of CRC cells to therapy through signaling pathways. MiRNA-506 was found to be low expressed in OXA-resistant CRC tissues. And the overexpression of miRNA-506 could not only inhibit the growth of CRC cells, but also reverse the resistance of OXA-resistant CRC cells to OXA. Further exploration by Zhou et al. (101) showed that miRNA-506 increased the sensitivity of CRC to OXA therapy by inhibiting MDR1/P-gp expression *via* downregulation of the Wnt/β-catenin pathway. miR-199b-3p was shown to enhance the drug resistance of CRC cells. Han et al. (102) suggested that suppressing miR-199b-3p could enhance the sensitivity of CRC cetuximab (CTx)-resistant cells to CTx *in vitro* and in mouse xenograft models by targeting the inhibition of CRIM1 *via* the Wnt/β-catenin signaling pathway. Wu et al. (103) found that the overexpression of miR-200b-3p could enhance OXA sensitivity in OXA-resistant CRC cells (HT29 and HCT116 cells) and induce growth inhibition and apoptosis of OXA-resistant CRC cells by inhibiting the expression of βIII-tubulin protein. As an important regulator of the PI3K/AKT signaling pathway, PTEN is also a direct target of miRNAs. The expression of miR-454-3p was significantly upregulated in OXA-resistant cells. Interestingly, inhibition of miR-454-3p was observed to sensitize OXA-resistant cells to OXA treatment and enhance OXA-induced cells apoptosis. In the xenograft model, this effect also exists. Meanwhile, Qian et al. (104) further confirmed that miR-454-3p enhanced OXA resistance by targeting PTEN and activating the AKT signaling pathway. And the PI3K/AKT signaling pathway is closely related to the chemosensitivity of CRC cells to 5-FU. Studies have shown that miRNAs affect the sensitivity of CRC cells to 5-FU by targeting the targets of the PI3K/AKT signaling pathway. Liu et al. (105) found that upregulation of miR-135b or miR-182 could enhance the resistance to 5-FU in CRC cells by targeting ST6GALNAC2 *via* the PI3K/AKT pathway. Similarly, Liu et al. (106) revealed that highly expressed miR-543 enhanced the resistance of CRC cells to 5-FU by downregulating the expression of PTEN, and the low expression of PTEN can activate the PI3K/AKT signaling pathway. As another regulator of the PI3K/AKT signaling pathway, PIK3R1 was confirmed to be a target of miR-455-5p in CRC cells. Lou et al. (107) found that miR-455-5p sensitized CRC cells to 5-FU through CCK-8 and flow cytometry analysis. They further studied the mechanism underlying this phenomenon and revealed that miR-455-5p enhanced the sensitivity of CRC cells to 5-FU by targeting PIK3R1 and DEPDC1. However, these conclusions were supported at the cellular level. And future *in vivo* studies to confirm these findings are urgently required. Shi et al. (108) selectively enriched cisplatin-resistant CRC cell lines from the SW480 cell line by using cisplatin. By PCR assay, they found that the expression of miR-148a was down-regulated in cisplatin-resistant SW480 cells, while overexpression of miR-148a was able to attenuate cisplatin resistance and inhibit the growth of CRC cells in cisplatin-resistant SW480 cells. Further research found that miR-148a played a role in regulating CRC cisplatin resistance and tumor development by inhibiting the expression of its downstream target Wnt10b and the activity of β-catenin signaling. And this conclusion was also validated in an immunized mouse xenograft model of SW480 resistance. The Notch pathway is a highly conserved signaling pathway that plays a key role in CRC, which contributes to cell proliferation, EMT and chemoresistance (117). MiR-139-5p was downregulated in 5-FU-resistant CRC cell lines (HCT-8/5-FU and HCT-116/5-FU). And the expression of miR-139-5p increased 5-FU-induced apoptosis and sensitized CRC cells to 5-FU by inhibiting NOTCH-1 and its downstream molecules (MRP-1 and BCL-2) (109). Similarly, in 5-FU-resistant cells (SW620 and HT-29 cells), the expression of miR-195-5p was decreased. Jin et al. (97) demonstrated that miR-195-5p reduced the stemness and chemoresistance of CRC cells by inhibiting the Notch signaling pathway.

The most common mechanism for cancer drug resistance is reduced drug accumulation concentrations in cancer cells, and studies have shown that a major cause of this phenomenon is increased drug efflux mediated by the ATP-binding cassette (ABC) efflux pump. ABC efflux pump can directly excrete drugs from cancer cells, affecting drug absorption, distribution, and metabolic clearance, resulting in chemotherapy failure (118, 119). Recently, studies on miRNAs have shown that miRNAs may regulate the drug resistance of CRC cells by affecting the ABC efflux pump. Liu et al. (110)showed that miR-128-3p inhibited EMT and increased intracellular OXA concentrations in OXA-resistant CRC cell lines. MiR-128-3p was found to competitively bind Bmi1 and MRP5 (an ABC efflux pump), resulting in decreased intracellular OXA efflux and enhanced OXA-induced EMT. Therefore, overexpression of miR-128-3p can enhance the therapeutic response of CRC cells to OXA.

Together, these studies provide a rationale for developing miRNA-based therapies to effectively treat drug-resistant CRC cells. However, one important limitation of these findings must be recognized. Most of the current research is at the cellular level and includes a few studies in animal models. There are more clinical research trials to be made before these miRNAs can truly become targeted drugs for reversing CRC resistance.

### Therapeutic approaches targeting miRNAs for CRC

As discussed above, some miRNAs promote CRC progression, metastasis, or drug resistance, while others show the opposite effect. According to their regulatory effects on tumors, miRNAs are broadly divided into two types: tumor suppressor miRNAs and oncogenic miRNAs. Therefore, the current treatment approaches based on targeting miRNAs include: (1) the upregulation of tumor suppressor miRNAs utilizing miRNA mimics, such as double-stranded synthetic miRNAs and miRNA expression vectors, when tumor suppressor miRNAs are downregulated; (2) inhibiting the expression of oncomiRs by miRNA antagonists, such as antisense oligonucleotides, antagomirs, and miRNA sponges, when oncomiRs are overexpressed (120). An effective treatment regimen requires not only the selection of the right regulatory molecules, but also the selection of appropriate drug delivery strategies. Although targeting miRNAs is a promising therapeutic strategy for the treatment of CRC, naked miRNA-based agents have many shortcomings, such as poor targeting capabilities, short circulation times, and off-target effects. In recent years, nanoparticle carriers have provided unprecedented opportunities for efficiently delivering therapeutics of miRNAs by controlling release kinetics, prolonging circulation time and improving biological distribution to improve the therapeutic efficacy of miRNA-targeting agents with less toxicity compared with other anticancer drugs (121, 122). For instance, miR-145 was shown to be downregulated in colon cancer and to have antiproliferative and proapoptotic effects. Through nanotechnology, Liang et al. (123) studied a PLGA/PEI-mediated miRNA vector delivery system and verified the validity of this method by using a colon cancer xenograft model with a miR-145 vector encoding for the expression of miR-145 (pDNA). The results of this work showed that miR-145 could be efficiently delivered to colon cancer cells and exerted potent antitumor efficacy through a PLGA/PEI/HA vehicle. Similarly, Ibrahim et al. (124) developed a miRNA delivery system by using polyethylenimine (PEI)-mediated delivery of unmodified miRNAs and validated the method in a mouse model of colon carcinoma. The results of this study showed that miRNA replacement therapy for miR-145 and miR-33a could reduce tumor growth. Despite the growing number of studies targeting miRNAs for the treatment of CRC, more studies, especially clinically relevant studies, are needed to demonstrate the clinical significance of therapeutic strategies for targeting miRNAs.

### Emerging role of circulating miRNAs as biomarkers in CRC

**Figure 2:**
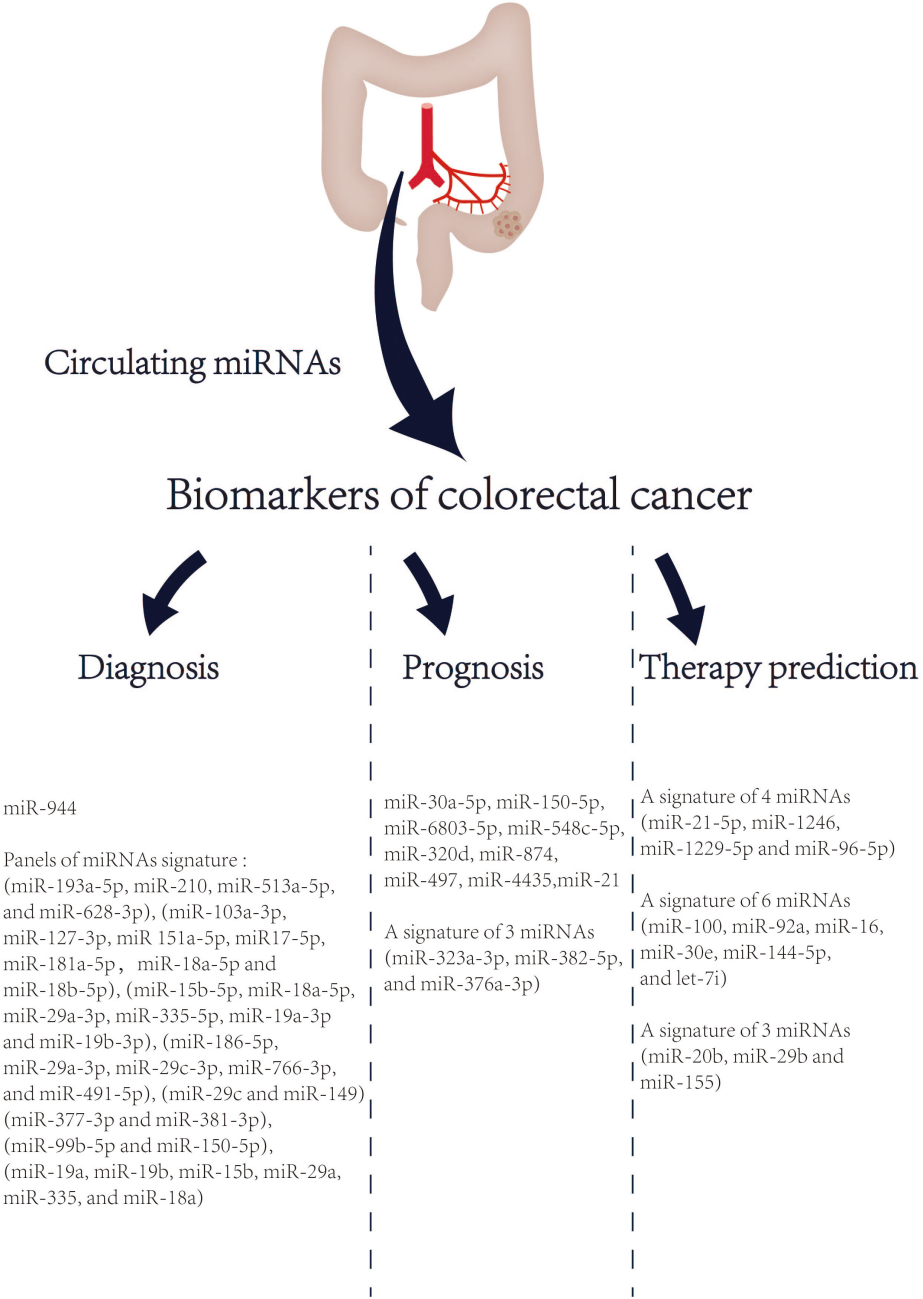
List of circulating miRNAs reported as promising biomarkers in CRC in recent 5 years. References are provided in Table S1.

Despite having a strong genetic component, most CRC cases are sporadic and undergo a lengthy (usually several years) process of slow progression from adenoma to cancer (125). The prognosis of CRC is highly related to the stage at diagnosis. The 5-year survival rate of early CRC patients can reach more than 90%, while the 5-year survival rate of advanced CRC patients is approximately 14% (2). Therefore, CRC screening and early diagnosis and treatment can effectively reduce the mortality of CRC. At present, the most frequently used diagnostic tool for CRC is colonoscopy. However, colonoscopy has some limitations in CRC screening, such as invasive procedures, poor population compliance, and high technical requirements for operators. In addition, CRC is a heterogeneous disease with various histologic characteristics, molecular characteristics, and prognosis. Although TNM staging system is a crucial clinical parameter to evaluate the prognosis of CRC patients, the prognosis of patients with CRC still varies considerably at the same stage (126). The drug resistance of CRC patients to current chemotherapy drugs also reflects its heterogeneity. Taken together, these limitations highlight the urgent need of CRC for new non-invasive biomarkers. MiRNA is relatively stable, not easily degraded by rnase, and less affected by high temperature and extreme pH. And it is a crucial regulator of life process, closely related to the tumor (can be actively secreted into the circulatory system by cancer cells) (127). Therefore, circulating miRNAs are a potential biomarker. In 2008, Mitchell et al. first proposed that circulating miRNAs are emerging as promising biomarkers for solid tumors (128). And then in 2009, Ng, EKO et al. (129) reported that circulating miRNA (MiR-92) was significantly increased in the plasma of CRC and might be a potential non-invasive biomarker for the diagnosis of CRC. In 2013, Kanaan, Ziad et al. (130) identified and verified the characteristics of miRNAs in the plasma of healthy controls, colorectal adenomas and CRC patients, and designed two powerful prediction models: a panel of 8 plasma miRNAs (miR-532-3p, miR-331, miR-195, miR-17, miR-142-3p, miR-15b, miR-532 and miR-652) could significantly distinguish colorectal adenomas from healthy people [AUC=0.868 (95% confidence interval [CI]: 0.76-0.98)]. Furthermore, a panel of 3 plasma miRNAs (miR-431, miR-15b and miR-139-3p) could also distinguish patients with stage IV CRC from healthy people [AUC=0.896 (95% CI: 0.78-1.0)]. Noteworthy, a growing number of studies have highlighted that circulating miRNAs, especially the prediction models based on miRNA panels, are a promising tool for early detection, prognosis and treatment selection of CRC in recent 5 years (**Figure 2**).

### Conclusion and future perspectives

In summary, miRNAs play important roles in CRC cell proliferation, metastasis, and chemoresistance by regulating CRC-related signaling pathways, EMT, angiogenesis and others. According to the different effects of miRNAs on tumors, miRNAs are also divided into tumor-promoting and tumor-suppressing types, and these two types also provide future directions for targeting miRNAs in the treatment of CRC. However, one of the greatest challenges in developing miRNA-based therapeutics is to design a delivery system that can make miRNA-based therapeutics durable and enable tissue-specific targeting while avoiding potential toxicities and off-target effects. Although there is no lack of scientific evidence that nanostructures containing miRNAs mimetics or antagonists can produce robust antitumor effects with few side effects, most of the current exciting results remain at the level of cell studies, and few relevant clinical studies have been reported. In conclusion, although miRNAs are a promising target for the treatment of CRC, the introduction of miRNA-targeted therapies into clinical practice still requires substantial and in-depth research.

## Author contributions

Literature review, data analysis, and manuscript preparation were performed by YY. W-JM and ZW contributed for the study conception, design, and revision. All authors contributed to the article and approved the submitted version.
