# Supplementary material for: MicroRNAs (miRNAs): Novel potential therapeutic targets in colorectal cancer
Source: Front Oncol. 2022 Dec 14;12:1054846. doi: 10.3389/fonc.2022.1054846 (PMC9794577; doi:10.3389/fonc.2022.1054846)
Supplement: Supplementary file 1 [file Table_1.docx]

**Table S1. List of references of Figure 2**

| **Circulating miRNAs** | **Biomarkers** | **References** |
| --- | --- | --- |
| A signature of 4 miRNAs (miR-193a-5p, miR-210, miR-513a-5p, and miR-628-3p) | Diagnosis | ^[1]^ |
| A signature of 7 miRNAs（miR-103a-3p、miR-127-3p、miR 151a-5p、miR17-5p、miR-181a-5p，miR-18a-5p and miR-18b-5p） | Diagnosis | ^[2]^ |
| A signature of 6 miRNAs (miR-15b-5p、miR-18a-5p、miR-29a-3p、miR-335-5p、miR-19a-3p and miR-19b-3p） | Diagnosis | ^[3]^ |
| A saliva-based miRNA signature (miR-186-5p, miR-29a-3p, miR-29c-3p, miR-766-3p, and miR-491-5p) | Diagnosis | ^[4]^ |
| miR-944 | Diagnosis | ^[5]^ |
| A signature of 2 miRNAs (miR-29c and miR-149） | Diagnosis | ^[6]^ |
| A signature of 2 miRNAs (miR-377-3p and miR-381-3p) | Diagnosis | ^[7]^ |
| A signature of 2 miRNAs (miR-99b-5p and miR-150-5p) | Diagnosis | ^[8]^ |
| A signature of 6 miRNAs (miR-19a, miR-19b, miR-15b, miR-29a, miR-335, and miR-18a) | Diagnosis | ^[9]^ |
| A signature of 3 miRNAs (miR-323a-3p, miR-382-5p, and miR-376a-3p) | Prognosis | ^[10]^ |
| miR-30a-5p | Prognosis | ^[11]^ |
| miR-150-5p | Prognosis | ^[12]^ |
| miR-6803-5p | Prognosis | ^[13]^ |
| miR-548c-5p | Prognosis | ^[14]^ |
| miR-320d | Prognosis | ^[15]^ |
| miR-874 | Prognosis | ^[16]^ |
| miR-497 | Prognosis | ^[17]^ |
| miR-4435 | Prognosis | ^[18]^ |
| miR-21 | Prognosis | ^[19]^ |
| A signature of 4 miRNAs (miR-21-5p, miR-1246, miR-1229-5p and miR-96-5p) | Therapy prediction | ^[20]^ |
| A signature of 6 miRNAs (miR-100, miR-92a, miR-16, miR-30e, miR-144-5p, and let-7i) | Therapy prediction | ^[21]^ |
| A signature of 3 miRNAs (miR-20b, miR-29b and miR-155) | Therapy prediction | ^[22]^ |

**References**

[1] Nakamura K, Hernández G, Sharma G G, Wada Y, Banwait J K, González N, et al. A Liquid Biopsy Signature for the Detection of Patients With Early-Onset Colorectal Cancer. Gastroenterology (2022) 163(5). doi:10.1053/j.gastro.2022.06.089

[2] Zhang H, Zhu M, Shan X, Zhou X, Wang T, Zhang J, et al. A panel of seven-miRNA signature in plasma as potential biomarker for colorectal cancer diagnosis. Gene (2019) 687: 246-54. doi:10.1016/j.gene.2018.11.055

[3] Marcuello M, Duran-Sanchon S, Moreno L, Lozano J J, Bujanda L, Castells A, et al. Analysis of A 6-Mirna Signature in Serum from Colorectal Cancer Screening Participants as Non-Invasive Biomarkers for Advanced Adenoma and Colorectal Cancer Detection. Cancers (Basel) (2019) 11(10). doi:10.3390/cancers11101542

[4] Rapado-González Ó, Majem B, Álvarez-Castro A, Díaz-Peña R, Abalo A, Suárez-Cabrera L, et al. A Novel Saliva-Based miRNA Signature for Colorectal Cancer Diagnosis. J Clin Med (2019) 8(12). doi:10.3390/jcm8122029

[5] Shaker O G, Ayeldeen G, Abdelhamid A M. Circulating microRNA-944 and its target gene EPHA7 as a potential biomarker for colorectal cancer. Arch Physiol Biochem (2022) 128(5): 1181-7. doi:10.1080/13813455.2020.1762658

[6] Abdul-Maksoud R S, Elsayed R S, Elsayed W S H, Sediq A M, Rashad N M, Shaker S E, et al. Combined serum miR-29c and miR-149 expression analysis as diagnostic genetic markers for colorectal cancer. Biotechnol Appl Biochem (2021) 68(4): 732-43. doi:10.1002/bab.1986

[7] Wang L, Song X, Yu M, Niu L, Zhao Y, Tang Y, et al. Serum exosomal miR-377-3p and miR-381-3p as diagnostic biomarkers in colorectal cancer. Future Oncol (2022) 18(7): 793-805. doi:10.2217/fon-2021-1130

[8] Zhao Y J, Song X, Niu L, Tang Y, Song X, Xie L. Circulating Exosomal miR-150-5p and miR-99b-5p as Diagnostic Biomarkers for Colorectal Cancer. Front Oncol (2019) 9: 1129. doi:10.3389/fonc.2019.01129

[9] Herreros-Villanueva M, Duran-Sanchon S, Martín A C, Pérez-Palacios R, Vila-Navarro E, Marcuello M, et al. Plasma MicroRNA Signature Validation for Early Detection of Colorectal Cancer. Clin Transl Gastroenterol (2019) 10(1): e00003. doi:10.14309/ctg.0000000000000003

[10] Lee I H, Kim G, Kwak S G, Baek D W, Kang B W, Kim H J, et al. Predictive Value of Circulating miRNAs in Lymph Node Metastasis for Colon Cancer. Genes (Basel) (2021) 12(2). doi:10.3390/genes12020176

[11] Sun Y, Yang B, Lin M, Yu H, Chen H, Zhang Z. Identification of serum miR-30a-5p as a diagnostic and prognostic biomarker in colorectal cancer. Cancer Biomark (2019) 24(3): 299-305. doi:10.3233/CBM-182129

[12] Zou S-L, Chen Y-L, Ge Z-Z, Qu Y-Y, Cao Y, Kang Z-X. Downregulation of serum exosomal miR-150-5p is associated with poor prognosis in patients with colorectal cancer. Cancer Biomark (2019) 26(1): 69-77. doi:10.3233/CBM-190156

[13] Yan S, Jiang Y, Liang C, Cheng M, Jin C, Duan Q, et al. Exosomal miR-6803-5p as potential diagnostic and prognostic marker in colorectal cancer. J Cell Biochem (2018) 119(5): 4113-9. doi:10.1002/jcb.26609

[14] Peng Z-Y, Gu R-H, Yan B. Downregulation of exosome-encapsulated miR-548c-5p is associated with poor prognosis in colorectal cancer. J Cell Biochem (2018). doi:10.1002/jcb.27291

[15] Tang Y, Zhao Y, Song X, Song X, Niu L, Xie L. Tumor-derived exosomal miRNA-320d as a biomarker for metastatic colorectal cancer. J Clin Lab Anal (2019) 33(9): e23004. doi:10.1002/jcla.23004

[16] Zhang N, Zhang P P, Huang J J, Wang Z Y, Zhang Z H, Yuan J Z, et al. Reduced serum exosomal miR-874 expression predicts poor prognosis in colorectal cancer. Eur Rev Med Pharmacol Sci (2020) 24(2): 664-72. doi:10.26355/eurrev_202001_20043

[17] Zou G, Wang R, Wang M. Clinical response and prognostic significance of serum miR-497 expression in colorectal cancer. Cancer Biomark (2019) 25(1): 11-8. doi:10.3233/CBM-181902

[18] Hong J W, Kim J M, Kim J E, Cho H, Kim D, Kim W, et al. MiR-4435 is an UQCRB-related circulating miRNA in human colorectal cancer. Sci Rep (2020) 10(1): 2833. doi:10.1038/s41598-020-59610-2

[19] Guraya S. Prognostic significance of circulating microRNA-21 expression in esophageal, pancreatic and colorectal cancers; a systematic review and meta-analysis. Int J Surg (2018) 60: 41-7. doi:10.1016/j.ijsu.2018.10.030

[20] Jin G, Liu Y, Zhang J, Bian Z, Yao S, Fei B, et al. A panel of serum exosomal microRNAs as predictive markers for chemoresistance in advanced colorectal cancer. Cancer Chemother Pharmacol (2019) 84(2): 315-25. doi:10.1007/s00280-019-03867-6

[21] Han J, Sun W, Liu R, Zhou Z, Zhang H, Chen X, et al. Plasma Exosomal miRNA Expression Profile as Oxaliplatin-Based Chemoresistant Biomarkers in Colorectal Adenocarcinoma. Front Oncol (2020) 10: 1495. doi:10.3389/fonc.2020.01495

[22] Ulivi P, Canale M, Passardi A, Marisi G, Valgiusti M, Frassineti G L, et al. Circulating Plasma Levels of miR-20b, miR-29b and miR-155 as Predictors of Bevacizumab Efficacy in Patients with Metastatic Colorectal Cancer. Int J Mol Sci (2018) 19(1). doi:10.3390/ijms19010307
